# U6 snRNA expression prevents toxicity in TDP-43-knockdown cells

**DOI:** 10.1371/journal.pone.0187813

**Published:** 2017-11-10

**Authors:** Masao Yahara, Akira Kitamura, Masataka Kinjo

**Affiliations:** Laboratory of Molecular Cell Dynamics, Faculty of Advanced Life Science, Hokkaido University, Sapporo, Japan; International Centre for Genetic Engineering and Biotechnology, ITALY

## Abstract

Depletion of amyotrophic lateral sclerosis (ALS)-associated transactivation response (TAR) RNA/DNA-binding protein 43 kDa (TDP-43) alters splicing efficiency of multiple transcripts and results in neuronal cell death. TDP-43 depletion can also disturb expression levels of small nuclear RNAs (snRNAs) as spliceosomal components. Despite this knowledge, the relationship between cell death and alteration of snRNA expression during TDP-43 depletion remains unclear. Here, we knocked down TDP-43 in murine neuroblastoma Neuro2A cells and found a time lag between efficient TDP-43 depletion and appearance of cell death, suggesting that several mechanisms mediate between these two events. The amount of U6 snRNA was significantly decreased during TDP-43 depletion prior to increase of cell death, whereas that of U1, U2, and U4 snRNAs was not. Downregulation of U6 snRNA led to cell death, whereas transient exogenous expression of U6 snRNA counteracted the effect of TDP-43 knockdown on cell death, and slightly decreased the mis-splicing rate of Dnajc5 and Sortilin 1 transcripts, which are assisted by TDP-43. These results suggest that regulation of the U6 snRNA expression level by TDP-43 is a key factor in the increase in cell death upon TDP-43 loss-of-function.

## Introduction

Transactivation response (TAR) RNA/DNA-binding protein 43 kDa (TDP-43) has been identified as an amyotrophic lateral sclerosis (ALS)-associated protein. TDP-43 is mainly localized in the nucleus and shuttles between the nucleus and cytoplasm to maintain several RNA-associated functions (e.g., local translation, translocation, splicing, and microRNA processing) [1]. However, in motor neurons from ALS patients, TDP-43 disappears from the nucleus and appears in cytoplasmic ubiquitinated inclusion bodies, along with carboxyl-terminal fragments (CTFs) of TDP-43 [2]. TDP-43 and TDP-43 CTFs are aggregation-prone and exert cytotoxicity in neuronal and non-neuronal cell lines [3–5]. Several groups including ours reported that RNA may be involved in the aggregation process of TDP-43 and TDP-43 CTFs [6–9]. Therefore, it is expected that toxic gain-of-function of RNA-involved aggregation of TDP-43 and TDP-43 CTFs may be implicated in neuronal cell death.

Alternatively, since TDP-43 knockout in murine motor neurons causes progressive motor neuron degeneration [10], loss-of-function of TDP-43 may be involved in ALS pathogenesis. TDP-43 knockout in mice exhibits early embryonic lethality [11–13]. Moreover, TDP-43 depletion in various mammalian cultured cells and embryonic stem cells results in cell death [14–17]. These results point at an essential role of TDP-43 in cell survival; however, the detailed mechanism of cell death during TDP-43 loss-of-function has not been elucidated.

TDP-43 depletion both in murine brain and mammalian cultured cells causes widespread alterations of the RNA-splicing state such as changes in exon inclusion [18–21]. Defects in RNA splicing are implicated in cell death in many neurodegenerative diseases including ALS [22,23]. These results imply that TDP-43 loss-of-function may cause cell death through alterations of the RNA-splicing state. An important machinery during RNA splicing in eukaryotes is the spliceosome, composed of small nuclear RNAs (snRNAs) including U1, U2, U4, U5, and U6 snRNA, in addition to a range of small nuclear ribonucleoproteins (snRNPs) [24]. Reports show that the expression profiles of such snRNAs are altered in TDP-43-knocked down cultured cells and spinal cord from ALS patients [25,26]. One study reported that the expression level of U6 snRNA was decreased in the spinal cord of ALS patients, but that of U1, U2, U4, and U5 snRNA was not [25]. Conversely, another study reported that the expression level of U6 snRNA in the spinal cord of ALS patients was not decreased, whereas that of U1, U2, U4, and U5 snRNA was increased [26]. Moreover, the amount of U6 snRNA in TDP-43-knocked down human neuroblastoma SH-SY5Y cells was not decreased in either study [25,26]. Thus, TDP-43 depletion can disturb expression of snRNAs; however, the expression profile of individual snRNAs during TDP-43 depletion remains unclear.

Therefore, to unveil the relationship between cell death and the alteration of snRNA expression during TDP-43 depletion, we investigated whether the expression level of U6 snRNA can be modified using TDP-43-knocked down murine neuroblastoma Neuro2A cells, which show a significant increase of cell death post-TDP-43 depletion. Finally, we investigated whether cell death during TDP-43 depletion can be prevented by restoring the expression levels of U6 snRNA.

## Material and methods

### Plasmid DNAs

Plasmid DNAs encoding GFP (pmEGFP-N1) and pmEGFP-N1 to express C-terminally GFP-tagged TDP-43 (TDP-43-GFP) were prepared as established previously [6]. Synthetic cDNA of TDP-43 that is not recognized by siRNA against murine TDP-43 mRNA was synthesized by Thermo Fisher Scientific (Waltham, MA). The cDNA was subcloned into pmEGFP-N1 to express C-terminally GFP-tagged TDP-43 (T43-GFP) according to a previous study [6]. Exogenous expression of U6 snRNA was driven by the human H1 promoter in the plasmid pSuperior.neo (Oligoengine, Seattle, WA). Synthetic U6 snRNA oligonucleotide was synthesized by Thermo Fisher Scientific (S1A Table) and subcloned into pSuperior.neo (pU6). Empty vector (pEV) was used as a negative control.

### Transfection and cell preparation

Murine neuroblastoma Neuro2A cells were kindly provided by Prof. Kazuhiro Nagata at Kyoto Sangyo University. The strain of the cell was as same as that in the previous studies, and culture conditions were reported previously [6,27]. For knockdown of TDP-43 and U6 snRNA, Neuro2A cells (5.5 × 10^5^) were plated in a 10 cm cell culture dish (CORNING, Corning, NY). A sense and antisense TDP-43-targeting siRNA duplex (T43-siRNA; 5’-GUUAGAAAGAAGUGGAAGATT-3’ and 5’-UCUUCCACUUCUUUCUAACTT-3’, respectively; synthesized by Nippon Gene, Tokyo, Japan), or a non-targeting siRNA (NC-siRNA; #AM4611; Thermo Fisher Scientific) as a negative control, were transfected using 24 μL Lipofectamine RNAiMAX transfection reagent (Thermo Fisher Scientific) and 160 pmol of each siRNA in Opti-MEM I medium (Thermo Fisher Scientific). A sense and antisense U6 snRNA-targeting siRNA duplex (U6-siRNA; 5’-GCUUCGGCAGCACAUAUACTT-3’ and 5’-GUAUAUGUGCUGCCGAAGCTT-3’, respectively; also synthesized by Nippon Gene) was modified from a previous study [28].

For expression of U6 snRNA in TDP-43-knocked down cells, Neuro2A cells (2.8 × 10^5^) were plated in a 10 cm culture dish. After a 20 h incubation, cells were transfected using 12.8 μL Lipofectamine 2000 transfection reagent (Thermo Fisher Scientific) and a mixture of 0.5 μg pmEGFP-N1 and either 4.5 μg pU6 or pEV. The transfection mix was incubated with the cells for 4 h. After the incubation, the medium was replaced and siRNA was transfected following the protocol described above (S1A and S1B Fig). Cell viability assays and Western blotting were performed using 3.5 cm culture dishes and by reducing the number of cells and amounts of plasmid DNA and reagents to 1/8 scale, accordingly.

### Western blotting

Recovery of cell lysates and Western blotting to detect the protein amount of TDP-43 and α-tubulin were performed as previously reported [6].

### PCR for non-coding RNAs or mRNAs

Total RNA was isolated with TRIzol (Thermo Fisher Scientific) and purified using PureLink RNA Mini Kit (Thermo Fisher Scientific) according to the manufacturer’s instructions. Extracts were treated with DNase I (TaKaRa, Shiga, Japan) before performing additional assays.

To detect non-coding RNAs, total RNA (750 ng) was used for synthesis of first-strand cDNA using Mir-X miRNA First Strand Synthesis Kit (TaKaRa) according to the manufacturer’s instructions. Real-time quantitative PCR (qPCR) was performed using SYBR Premix Ex Taq (Tli RNase H Plus; TaKaRa), ROX Reference Dye II (TaKaRa), 0.2 μM forward primer, 0.2 μM reverse primer, and 2 μL template cDNA (corresponding to 15 ng total RNA). Amplification and detection were performed using a real-time PCR system (Mx3005P; Agilent Technologies, Santa Clara, CA). The PCR was performed in a two-step protocol under the following conditions: initial denaturing at 95°C for 30 s, followed by 35 cycles of denaturing at 95°C for 10 s, and annealing/extension at 60°C for 20 s.

To detect the splicing state of transcripts, first-strand complementary DNA synthesis was performed using a transcriptase (PrimeScript; TaKaRa) according to the manufacturer’s instructions. PCR was performed using a thermal cycler (Bioer Technology, Binjiang, China). The PCR was performed in a three-step protocol under the following conditions: initial denaturing at 98°C for 30 s, followed by 21 cycles of denaturing at 98°C for 10 s, annealing at 60°C for 30 s, and extension at 72°C for 60 s. PCR products were separated in 5% polyacrylamide gel and detected using LAS 4000 mini (Fujifilm, Tokyo, Japan). PCR primers are described in S1B Table.

### Urea-polyacrylamide gel electrophoresis (Urea-PAGE) for RNA

Samples including total extract RNA (2.75 μg) were mixed with loading buffer consisting of 1.5× Tris-acetate EDTA (TAE), 10 M urea, 10% (w/v) sucrose, 0.05% (w/v) bromophenol blue, and 0.05% (w/v) xylene cyanol, incubated at 65°C for 15 min, and kept on ice until electrophoretic separation. Samples were separated in a 12% polyacrylamide gel containing 7% urea in 1× TAE buffer. RNAs were stained using SYBR Gold (Thermo Fisher Scientific). Fluorescent intensities were detected using LAS 4000 mini (Fujifilm) using a 460 nm excitation light and a long-pass filter (Y515; Fujifilm). Quantification was performed using ImageJ 1.47 software (National Institutes of Health, Bethesda, MD).

### Cell viability assay

Dead cells were stained by 1.0 μg/mL propidium iodide (PI; Sigma-Aldrich, St. Louis, MO) and observed using an LSM 510 (Carl Zeiss, Jena, Germany) and a Plan-Neofluar 10×/0.3 NA objective as reported previously [6,27].

For alternative cell viability assays based on metabolism, at 96 h after siRNA transfection, cells (0.5 × 10^5^) in 100 μL cell culture medium were re-plated into a 96-well plate (Asahi Technoglass, Shizuoka, Japan) and cultivated for 20 h. Thereafter, 10 μL WST-1 mixture (Roche, Basel, Switzerland) was added to the cell culture medium, samples were incubated for an additional 4 h, and then the medium was collected. Absorbance at 490 and 690 nm was measured using a DU 800 spectrophotometer (Beckman Coulter, Brea, CA). Absorbance at 490 nm was subtracted from that at 690 nm.

### Cross-linking immunoprecipitation (CLIP) assay

Neuro2A cells were passaged into four 10 cm culture dishes at a ratio of 1/5 and transfected using 16.0 μL Lipofectamine 2000 transfection reagent and 4.0 μg pmEGFP-N1 or TDP-43-GFP expression plasmid (TDP-43-GFP). After incubation for 30 h, the CLIP assay was performed as previously reported [29]. Cells were fixed in phosphate-buffered saline containing 3% formaldehyde and lysed by sonication. Immunoprecipitation was performed overnight at 4°C using anti-GFP monoclonal antibody-conjugated agarose beads (MBL, Nagoya, Japan). After dissociation of cross-linked complexes at 70°C, RNA was extracted, reverse-transcribed, and U6 snRNA transcripts were quantified by qPCR as described in the “PCR for non-coding RNAs or mRNAs” sub-section.

### Statistics

Student’s *t* test was performed to evaluate statistical significance.

## Results

### Temporal delay between TDP-43 depletion and cell death

**Fig 1:**
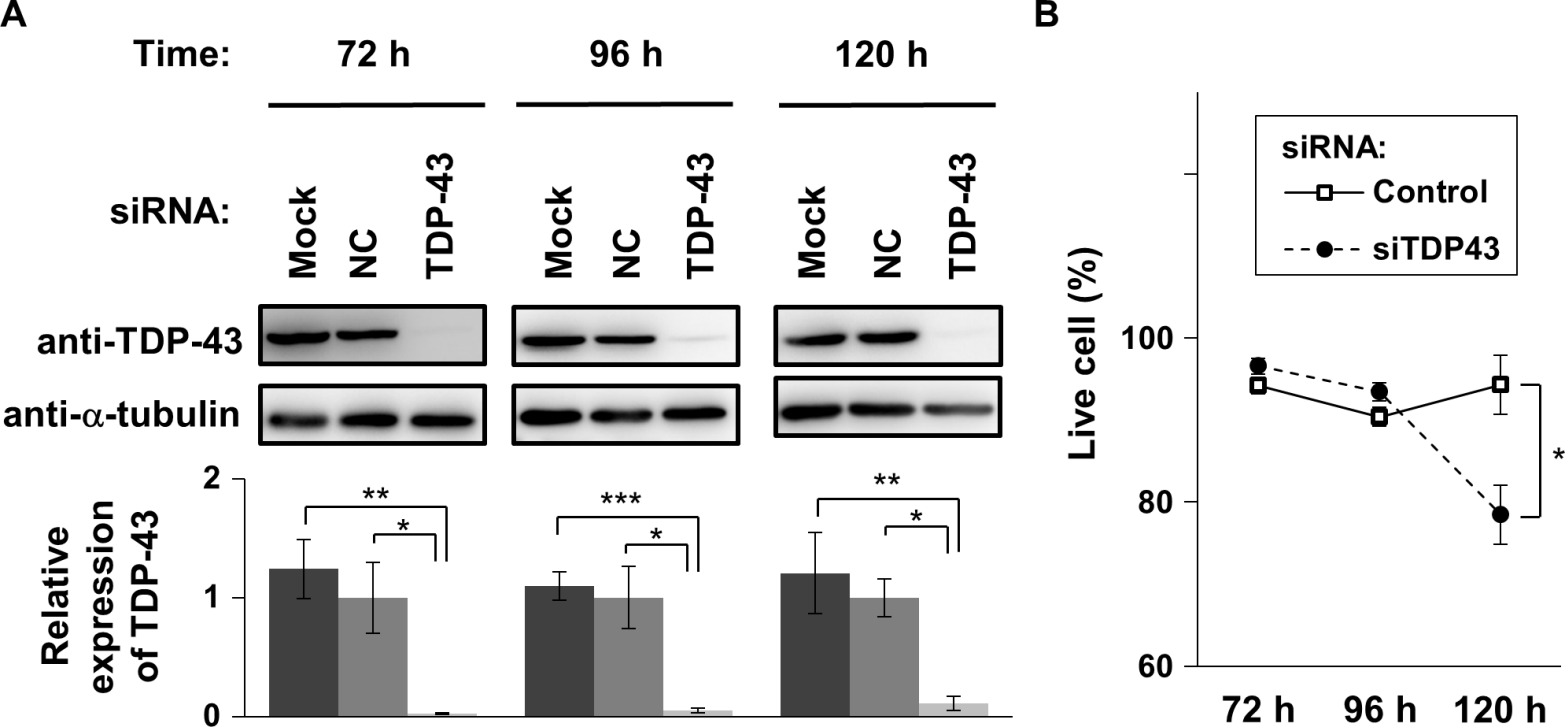
Time course of cell death during TDP-43 depletion. (A) Western blot analysis of endogenous protein expression of TDP-43 and α-tubulin during siRNA-mediated TDP-43 knockdown. Time after transfection of T43-siRNA, NC-siRNA (NC), or no siRNA and transfection reagents (Mock) is indicated (72, 96, and 120 h). α-tubulin was used to normalize band intensities of TDP-43. Bars in bottom graphs indicate the normalized amount of endogenous TDP-43 (mean ± SEM; n = 3). Significance between indicated pairs was tested by Student’s *t* test: *p < 0.05, **p < 0.01, and ***p < 0.001. (B) The proportion of live cells at 72, 96, and 120 h after transfection of T43-siRNA and NC-siRNA as a negative control (circle and square, respectively; mean ± SEM; n = 3). Significance between T43-siRNA- and NC-siRNA-transfected cells was tested by Student’s *t* test: *p < 0.05.

We first sought to evaluate the timeline of TDP-43 depletion following knockdown. To knock down TDP-43, siRNA targeting TDP-43 mRNA (T43-siRNA) or non-coding siRNA (NC-siRNA) were transiently transfected into Neuro2A cells. TDP-43 protein levels were assessed by Western blot at 72, 96, and 120 h after transfection. T43-siRNA-transfected cells showed a significant decrease in TDP-43 levels compared with NC-siRNA-transfected cells. In turn, NC-siRNA transfection did not have any significant effect on the levels of TDP-43 compared with mock-transfected cells. These results indicate that T43-siRNA-mediated knockdown can achieve an efficient reduction in the protein levels of TDP-43 (Fig 1A). Moreover, the protein levels of TDP-43 remained low (<10% of control) until at least 120 h after siRNA transfection (Fig 1A).

Next, we analyzed the timeline of cell death after TDP-43 depletion. Quantification of cell death showed that the proportion of live cells remained stable at 72 and 96 h after transfection with siRNA and significantly decreased at 120 h after siRNA transfection (Fig 1B). Given that the protein level of TDP-43 was significantly decreased at 72 and 96 h after transfection (Fig 1A), whereas the proportion of live cells did not significantly decrease until 120 h after siRNA treatment (Fig 1B), these results indicate that there is a time lag between TDP-43 depletion and the constitution of cell death phenotype.

### U6 snRNA is downregulated in TDP-43-knocked down cells

**Fig 2:**
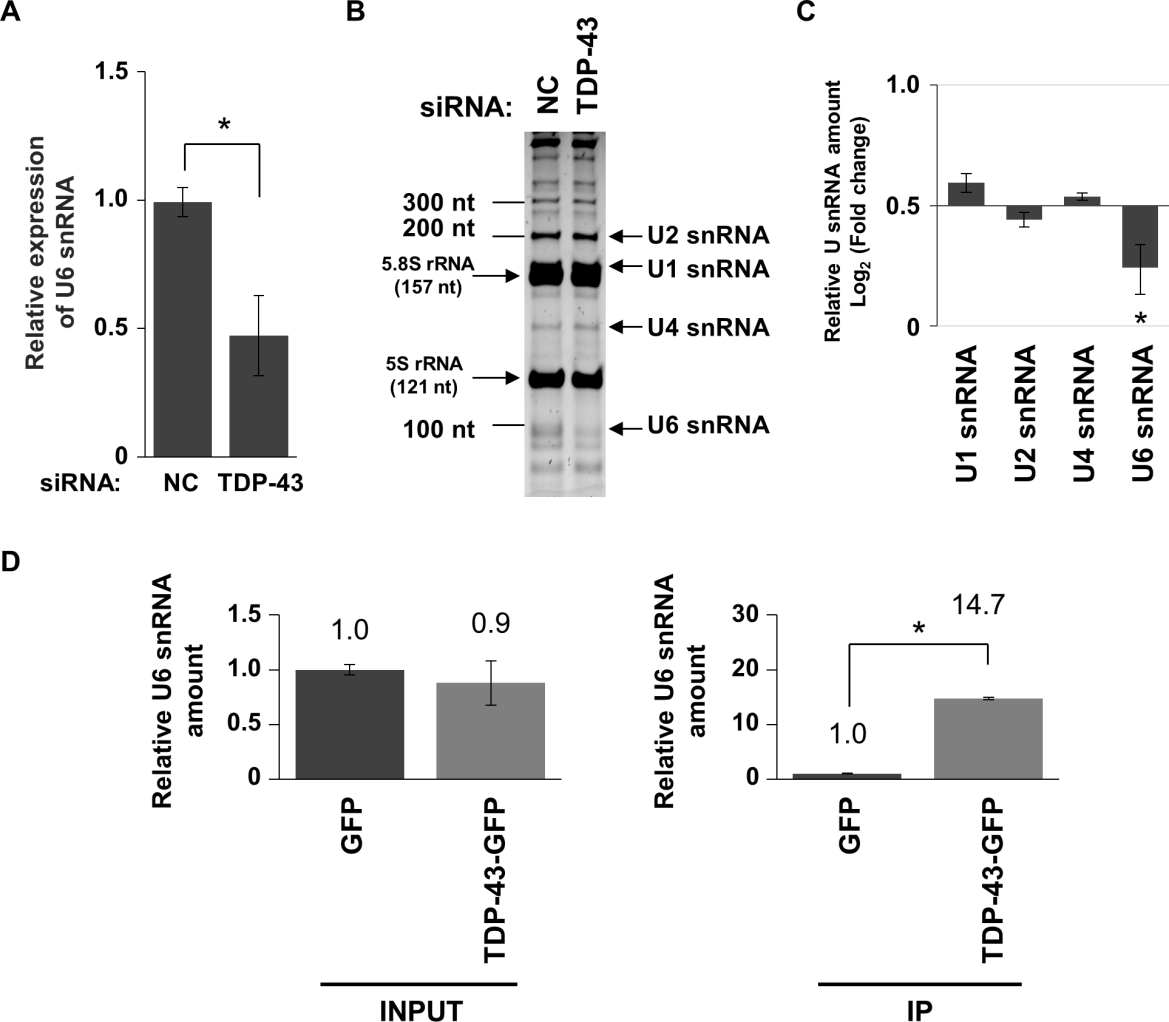
Downregulation of U6 snRNA in TDP-43-knocked down Neuro2A cells. (A–C) Quantification of the expression change of small RNAs during TDP-43 knockdown. Significance was tested by Student’s *t* test: *p < 0.05. (A) Relative expression of U6 snRNA in T43-siRNA-transfected cells compared with that in NC-siRNA-transfected cells. The expression level of 18S rRNA was used as an internal control (mean ± SEM, n = 3). (B) Gel image of Urea-PAGE. Left bar indicates molecular size in nucleotides (nt) of marker. The two dominant bands correspond to 5.8S and 5S ribosomal RNA (rRNA). (C) Relative amount of U1, U2, U4, and U6 snRNAs in T43-siRNA-transfected cells compared with that in NC-siRNA-transfected cells (mean ± SEM, n = 3). (D) Quantification of the binding of U6 snRNA to TDP-43 by CLIP. The amount of U6 snRNA in the sample containing TDP-43-GFP was normalized against that in the sample containing GFP as a negative control in each input cell lysate (INPUT) or immunoprecipitated mixture (IP) (mean ± SEM; n = 3). Significance indicated in the graph was tested by Student’s *t* test: *p < 0.05.

Next, we checked the expression level of U6 snRNA in TDP-43-knocked down Neuro2A cells at 72 h after the transfection of siRNAs using qPCR. The amount of U6 snRNA in T43-siRNA-transfected cells was significantly decreased compared with that in NC-siRNA-transfected cells (S2 Fig). The amount of 18S ribosomal RNA (18S rRNA) and 7SL RNA, ubiquitously expressing small RNAs transcribed from housekeeping genes, was not changed by TDP-43 knockdown, but that of small nucleolar RNA 202 (snoRNA202) was increased, indicating that 18S rRNA and 7SL RNA can be used as an internal control during TDP-43 depletion (S2 Fig). Thus, to evaluate the amount of U6 snRNA in TDP-43-knocked down cells, we used 18S rRNA as an internal control. As a result, the expression level of U6 snRNA in TDP-43-knocked down cells was decreased to approximately 50% of that in NC-siRNA-transfected cells (Fig 2A).

We next elucidated the expression levels of other U snRNAs using urea-polyacrylamide gel electrophoresis (Urea-PAGE). The migration pattern of U1, U2, U4, and U6 snRNA was unequivocally assigned using their predicted molecular sizes (164, 187, 145, and 107 nucleotides, respectively). Unfortunately, U5 snRNA was not distinguished in this migration pattern (Fig 2B). The decreased amount of U6 snRNA during TDP-43 depletion was consistent with the results obtained using qPCR, whereas the amount of U1, U2, and U4 snRNA was not changed (Fig 2C). These results suggest that U6 snRNA is selectively downregulated during TDP-43 depletion in Neuro2A cells.

Moreover, to investigate whether TDP-43 can associate with U6 snRNA, we quantified the amount of U6 snRNA co-immunoprecipitated with TDP-43 using qPCR. The amount of U6 snRNA did not differ between lysates of TDP-43-GFP-expressing and GFP-expressing cells (Fig 2D; INPUT). However, the level of U6 snRNA co-precipitated with TDP43-GFP was 14.7-fold higher than that co-precipitated with GFP (Fig 2D; IP). Thus, TDP-43 may directly bind to U6 snRNA and thereby regulate its stability.

### Downregulation of U6 snRNA results in cell death

**Fig 3:**
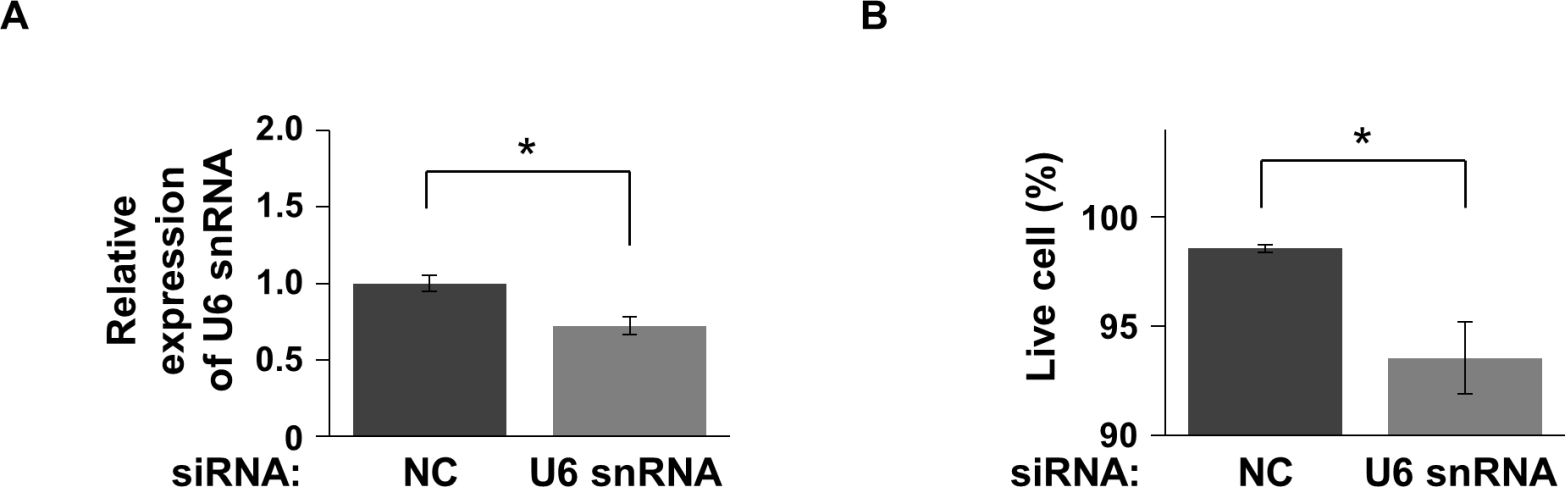
Viability of U6 snRNA-depleted cells. (A) Quantification of U6 snRNA expression at 72 h after transfection of U6-siRNA and NC-siRNA. The expression level of 18S rRNA was used as an internal control (mean ± SEM, n = 3). Significance was tested by Student’s *t* test: *p < 0.05. (B) The proportion of live cells at 72 h after transfection of U6-siRNA and NC-siRNA (mean ± SEM; n = 3). Significance was tested by Student’s *t* test: *p < 0.05.

To investigate whether U6 snRNA is involved in cell death, U6 snRNA was knocked down using siRNA. At 72 h after transfection of U6-siRNA, U6 snRNA expression in Neuro2A cells was decreased to 72% compared with that in NC-siRNA-transfected cells (Fig 3A). The proportion of live cells was significantly decreased upon U6 snRNA knockdown (Fig 3B), indicating that downregulation of U6 snRNA increases cell death. Thus, downregulation of U6 snRNA during TDP-43 depletion may contribute to cell death. The proportion of dead cells was higher upon TDP-43 knockdown (21%, Fig 1B) than upon U6 snRNA knockdown (6.5%, Fig 3B). This may be due to a difference in the degree to which U6 snRNA was downregulated (28% upon U6 snRNA knockdown and 53% upon TDP-43 knockdown).

### Expression of U6 snRNA prevents cell death during TDP-43 depletion

**Fig 4:**
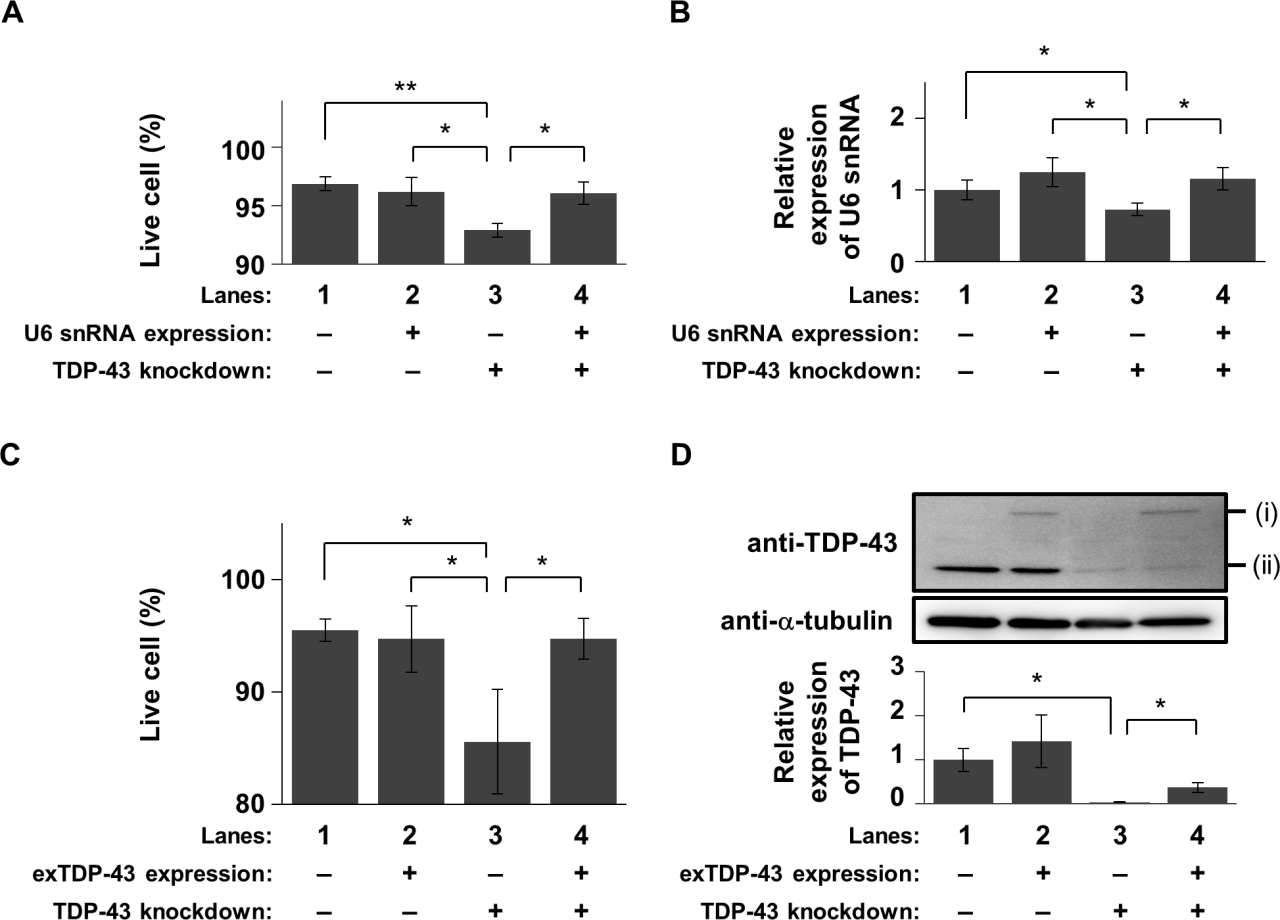
Amelioration of cell death by transient expression of U6 snRNA in TDP-43-knocked down cells. Significance indicated in the graph was tested by Student’s *t* test: *p < 0.05 and **p < 0.01. (A) The proportion of live cells at 120 h after transfection of T43-siRNA or NC-siRNA among U6 snRNA-expressing or non-expressing cells (mean ± SEM; n = 4). (B) Quantification of expression of U6 snRNA using qPCR at 72 h after transfection of U6 snRNA expression plasmid DNA or empty vector (mean ± SEM; n = 4). (C) The proportion of live cells at 120 h after transfection of T43-siRNA or NC-siRNA among TDP-43-expressing or control cells (mean ± SEM; n = 4). (D) Protein abundance of TDP-43 and α-tubulin during transient expression of T43-GFP for 76 h. Top: Western blotting using an anti-TDP-43 or anti-α-tubulin antibody. Bands (i) and (ii) indicate T43-GFP and endogenous TDP-43, respectively. Bottom: Quantification of relative expression of TDP-43 (mean ± SEM; n = 4).

To determine whether restoration of U6 snRNA expression rescues cell death during TDP-43 depletion, we examined cell viability when exogenous U6 snRNA was transiently expressed using a plasmid DNA encoding U6 snRNA (pU6) in T43-siRNA-transfected Neuro2A cells. Transfection with an empty vector (pEV) as a negative control decreased the proportion of live cells during TDP-43 knockdown at 120 h (Fig 4A, lanes 1 & 3). Interestingly, the proportion of live cells during TDP-43 knockdown was significantly restored by transfection of pU6 (Fig 4A, lanes 3 & 4). Transfection of pU6 in NC-siRNA-transfected cells did not change the proportion of live cells (Fig 4A, lanes 1 & 2). Another assay based on cellular metabolism also demonstrated the recovery of cell viability by transfection of pU6 during TDP-43 knockdown (S3 Fig).

Next, to confirm whether transfection of pU6 effectively increased the amount of U6 snRNA, we performed a qPCR at 76 h after transfection with pU6. The amount of U6 snRNA in NC-siRNA-transfected cells showed a slight increase by transfection with pU6, but the difference was not significant (Fig 4B, lanes 1 & 2). A possible reason for the slight increase of U6 snRNA expression may be autoregulatory transcriptional repression during over-expression of U6 snRNA [16]. However, in the case of TDP-43 knockdown, the amount of U6 snRNA was significantly higher in pU6-transfected cells than in control pEV-transfected cells (Fig 4B, lanes 3 & 4). We therefore concluded that transient rescue of U6 snRNA expression during TDP-43 depletion can be achieved. Consequently, restoration of U6 snRNA expression efficiently slowed down the constitution of cell death phenotype during TDP-43 depletion.

We confirmed that the proportion of live cells among T43-siRNA-transfected cells was restored by exogenous expression of a TDP-43 that is resistant to knockdown by T43-siRNA. Expression of exogenous TDP-43 tagged with GFP (T43-GFP) in NC-siRNA-transfected cells did not alter the proportion of live cells (Fig 4C, lanes 1 & 2). Expression of T43-GFP in T43-siRNA-transfected cells increased the proportion of live cells (Fig 4C, lanes 3 & 4). In this case, expression of T43-GFP was confirmed at 72 h after transfection (Fig 4D). Quantification of protein levels indicated that transfection of exogenous TDP-43 resulted in a 33.2% ± 9.8% recovery of TDP-43 protein levels compared with the endogenous levels (Fig 4D, lanes 1 & 4). The 33.2% abundance of TDP-43 may be sufficient to prevent to cell death.

In addition, exogenous expression of U6 snRNA in TDP-43-knocked down cells did not alter the amount of endogenous TDP-43 until 120 h after transfection of T43-siRNA (S4A and S4B Fig). Therefore, U6 snRNA may have a protective role in cell death during TDP-43 depletion.

### Exogenous expression of U6 snRNA partially ameliorates mis-splicing during TDP-43 depletion

**Fig 5:**
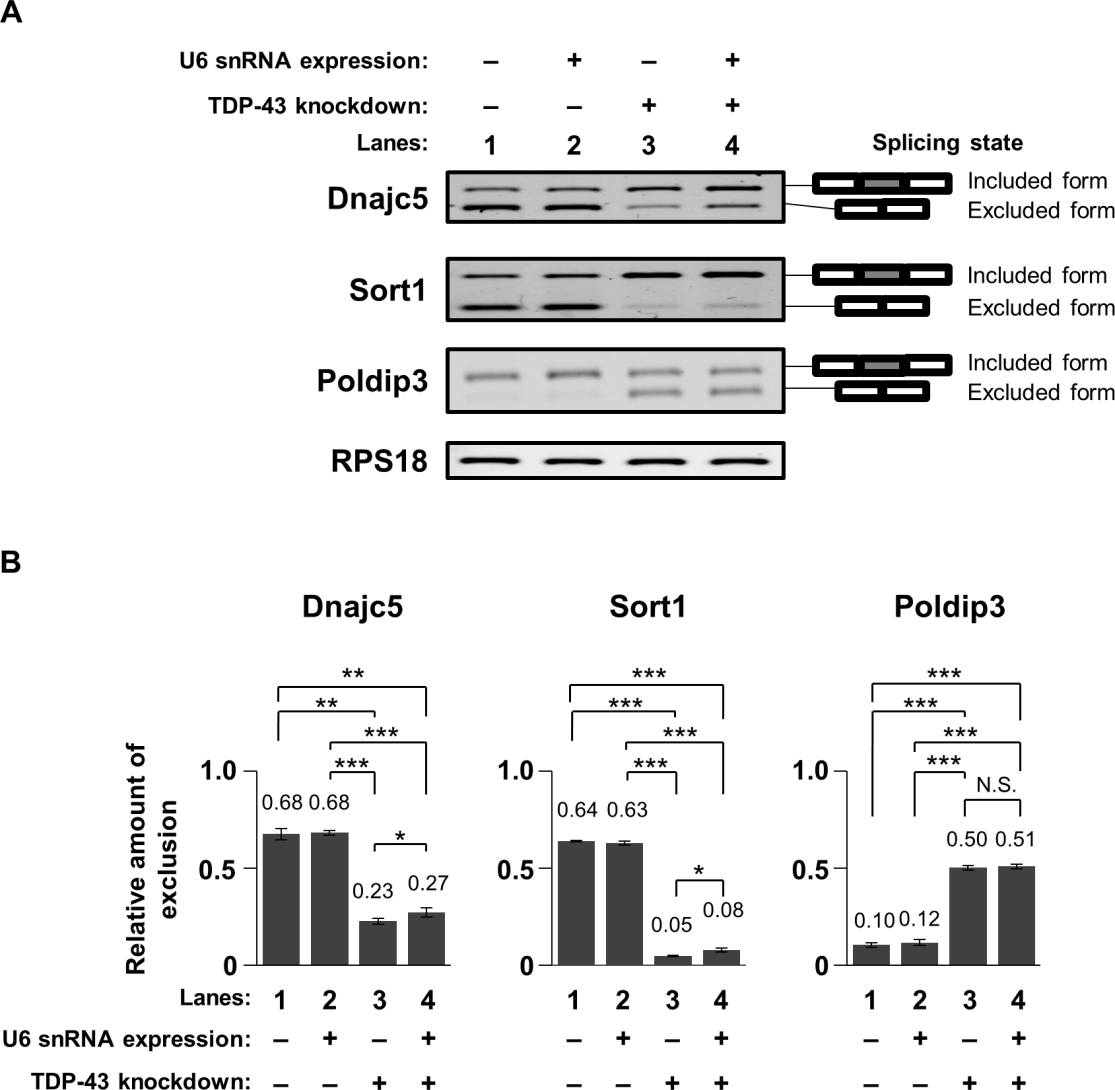
Change in the exon-exclusion state of transcripts during expression of U6 snRNA in TDP-43-knocked down cells. (A) Images of migrated bands of spliced forms of Dnajc5, Sort1, and Poldip3 transcripts. RPS18 was used as an internal loading control. Spliced forms of transcripts were distinguished using splicing-dependent pairs of PCR primers. (B) Relative amounts of the exon-excluded forms of Dnajc5, Sort1, and Poldip3 transcripts (mean ± SEM; n = 5). Significance was tested by Student’s *t* test: *p < 0.05, **p < 0.01, and ***p < 0.001. Numbers in each bar show mean values.

To investigate a plausible mechanism by which an increase in the U6 snRNA level ameliorates the viability of TDP-43-knocked down cells, we examined the aberrant mRNA-splicing state during TDP-43 depletion. Based on previous reports [18–20, 30], we selected four transcripts (Dnajc5, Sortilin 1 [Sort1], Poldip3, and Madd) as mis-splicing targets during TDP-43 depletion. The splicing of these transcripts was altered in TDP-43-knocked down cells (Fig 5A, lanes 1 & 3 and S5A Fig, lanes 1 & 3).

Next, we analyzed whether expression of exogenous U6 snRNA changes the amount of the spliced form of these transcripts in TDP-43-knocked down cells. The amount of the exon-excluded form of Dnajc5 and Sort1 transcripts was slightly increased by exogenous expression of U6 snRNA in TDP-43-knocked down cells (Fig 5B, lanes 3 & 4), but not in NC-siRNA-transfected cells (Fig 5B, lanes 1 & 2), whereas the splicing of Poldip3 and Madd transcripts was not changed by exogenous expression of U6 snRNA in TDP-43-knocked down cells (Fig 5B and S5B Fig). These results suggest that the splicing efficiency of Dnajc5 and Sort1 transcripts in TDP-43-knocked down cells can be partially restored by exogenous expression of U6 snRNA. Moreover, it is possible that U6 snRNA may have a protective role against the alteration of splicing efficiency of a portion of transcripts during TDP-43 depletion.

## Discussion

We show here that TDP-43 knockdown downregulates the amount of U6 snRNA but not of U1, U2, and U4 snRNAs in Neuro2A cells. Two possible mechanisms could explain this downregulation: TDP-43 could be involved in (i) transcriptional promotion *via* RNA polymerase III and/or (ii) stability of U6 snRNA. In the first case, U6 snRNA is transcribed by RNA polymerase III, while U1, U2, and U4 snRNAs are transcribed by RNA polymerase II [24]. However, while 7SL RNA is also transcribed by RNA polymerase III [31], our results show that 7SL RNA levels were not decreased during TDP-43 depletion (S2 Fig). Therefore, transcriptional promotion *via* RNA polymerase III may not contribute to the decrease of U6 snRNA levels during TDP-43 depletion. In the second case, the U6 snRNA sequence does not contain typical UG repeats, which TDP-43 preferably recognizes; however, CLIP showed that a low amount of U6 snRNA binds to TDP-43 [19]. We confirmed that U6 snRNA co-precipitated with TDP-43-GFP (Fig 2D), demonstrating that TDP-43 can associate with U6 snRNA. In addition, proteomic analysis revealed that TDP-43 interacts with SART1 and PRPF3, two components of U6 snRNA-containing complexes [32]. These findings suggest that the depletion of TDP-43 could destabilize the complex, releasing free U6 snRNA, which is likely degraded. Therefore, TDP-43 may control the stability of U6 snRNA by directly maintaining the RNA-protein complex and not *via* an intermediate process (e.g., an increased level of ribonuclease for U6 snRNA during TDP-43 depletion). However, it is unknown whether TDP-43 binds to U6 snRNA directly or *via* other proteins, and this should be clarified in a further study.

Although a previous study using human SH-SY5Y cells reported that the amount of U6 snRNA was not decreased by TDP-43 knockdown [25,26], we show that U6 snRNA in TDP-43-knocked down murine Neuro2A cells is downregulated (Fig 2). This discrepancy is likely due to differences in the species and/or cell lines, since the expression profile of U snRNAs after siRNA-mediated knockdown in survival motor neurons protein (SMN) differs between human and murine cell lines [33]. However, we show that expression of exogenous U6 snRNA can efficiently rescue cell death during TDP-43 depletion (Fig 4). These results suggest that downregulation of U6 snRNA is involved in the death of TDP-43-knocked down murine Neuro2A cells.

In addition, our results show that expression of U6 snRNA in TDP-43-knocked down cells slightly improved the mis-splicing efficiency of Dnajc5 and Sort1 transcripts. Although the direct effect of U6 snRNA on the alternative splicing of transcripts in the TDP-43-knockdown condition has not been clarified yet, we expect that restoration of U6 snRNA levels during TDP-43 depletion might maintain the alternative splicing efficiency of these transcripts. On the other hand, TDP-43 loss-of-function inhibits vesicular trafficking, including endosomal trafficking and autophagosome-lysosome fusion [15,34]. These defects may contribute to cell death by altering the trophic state. Restoration of U6 snRNA expression following TDP-43 depletion may suppress cell death by regulating the expression of genes important for trophic signaling. Future studies should explore the detailed mechanisms by which downregulation of U6 snRNA expression affects alternative splicing and increases cell death upon TDP-43 depletion.

Although our results are confined to the cellular level, a report showed a decrease in the U6 snRNA levels in spinal cord from ALS patients [26]. Therefore, we expect that downregulation of U6 snRNA might be involved in loss-of-function of TDP-43 in ALS pathophysiology and correction of U6 snRNA expression might slow down neuronal cell death during TDP-43 loss-of-function in ALS patients. Thus, investigations of an endogenous mechanism and/or drug to maintain the expression levels of U6 snRNA during loss-of-function of TDP-43 would lead to the elucidation of ALS pathophysiology.

## Supporting information

S1 Table List of synthetic oligonucleotides and PCR primers. (A) Synthetic oligonucleotides used to construct the U6 snRNA expression plasmid. (B) PCR primers used to detect transcripts and small RNAs. † denotes a commercially available primer included in the Mir-X First Strand Synthesis Kit (TaKaRa).
(PDF)

S1 Fig Schematic of the time course of transfection. (A) Transfection of T43-siRNA, total RNA extraction, protein extraction, and staining of dead cells. Green indicates the period of siRNA transfection. (B) Transfection of U6 snRNA plasmid DNA prior to transfection of T43-siRNA, total RNA extraction, protein extraction, and staining of dead cells. Blue and green indicate the period of plasmid DNA and siRNA transfection, respectively.
(TIF)

S2 Fig Fold changes in PCR-amplified amounts of small RNAs during TDP-43 knockdown. Relative PCR-amplified amounts of U6 snRNA, 18S rRNA, 7SL RNA, and snoRNA 202 in T43-siRNA-transfected cells compared with those in NC-siRNA-transfected cells at 72 h after siRNA transfection (mean ± SEM; n = 3). Significance was tested by Student’s *t* test: *p < 0.05.
(TIF)

S3 Fig Quantification of the viability of TDP-43-knocked down cells transfected with U6 snRNA by the WST-1 assay. Absorbance at 450 nm was subtracted from that at 690 nm. Cells were cultured for 120 h after transfection of TDP-43-siRNA or NC-siRNA, with or without exogenous U6 snRNA expression. Significance indicated in the graph was tested by Student’s *t* test: *p < 0.05 and ***p < 0.001 (mean ± SEM; n = 5).
(TIF)

S4 Fig Time course of TDP-43 expression during transient U6 snRNA expression. (A) Western blot analysis of TDP-43 and α-tubulin in T43-siRNA- or NC-siRNA-transfected cells transiently expressing U6 snRNA. Time corresponds to the amount of time after siRNA transfection. (B) Quantification of TDP-43 expression by Western blotting using anti-TDP-43 and anti-α-tubulin antibodies (mean ± SEM; n = 3). Significance indicated in the graph was tested by Student’s *t* test: *p < 0.05. N.S. denotes no statistical significance.
(TIF)

S5 Fig Change in the splicing of Madd transcripts during expression of U6 snRNA in TDP-43-knocked down cells. (A) Images of migrated bands of spliced forms of Madd transcripts. RPS18 was used as an internal loading control. Spliced forms of transcripts were distinguished using splicing-dependent pairs of PCR primers. (B) Relative amount of the exon-excluded form of Madd transcripts (mean ± SEM; n = 5). Significance was tested by Student’s *t* test: *p < 0.05 and ***p < 0.001. N.S. denotes no statistical significance. Numbers in each bar show mean values.
(TIF)
