# Supplementary material for: U6 snRNA expression prevents toxicity in TDP-43-knockdown cells
Source: PLoS One. 2017 Nov 10;12(11):e0187813. doi: 10.1371/journal.pone.0187813 (PMC5681290; doi:10.1371/journal.pone.0187813)
Supplement: S1 Table — (A) Synthetic oligonucleotides used to construct the U6 snRNA expression plasmid. (B) PCR primers used to detect transcripts and small RNAs. † denotes a commercially available primer included in the Mir-X First Strand Synthesis Kit (TaKaRa). (PDF) [file pone.0187813.s001.pdf]

A

Synthetic oligonucleotide for U6 snRNA expression plasmid	
Sense	5'-AGATCCCCGTGCTCGCTTCGGCAGCACATATACTAAAATTGGAACGATACAGAGAAGATTTAGCATGGCCCCTGCGCAAGGATGACACGCAAATTCGTGAAGCGTTCCATATTTTTAAGCTT-3'
Antisense	5'-AAGCTTAAAAATATGGAACGCTTCACGAATTTGCGTGTCATCCTTGCGCAGGGGCCATGCTAAATCTTCTCTGTATCGTTCCAATTTTAGTATATGTGCTGCCGAAGCGAGCACGGGGATCT-3'

B

Primers for PCR		
Transcript name	Forward primer	Reverse primer
U6 snRNA	5'-CTCGCTTCGGCAGCACATATACT-3'	5'-ACGCTTCACGAATTTGCGTGTC-3'
RPS18	5'-GGGCGGAGATATGCTCATGTG-3'	5'-TCTGGGATCTTGTACTGTCGT-3'
7SL RNA	5'-GGAGTTCTGGGCTGTAGTGC-3'	5'-ATCAGCACGGGAGTTTTGAC-3'
18S rRNA	5'-GTAACCCGTTGAACCCCATT-3'	5'-CCATCCAATCGGTAGTAGCG-3'
snoRNA 202	5'-AGTACTTTTGAACCCTTTTCC-3'	†Commercial primer
Sort1	5'-CAGGAGACAAATGCCAAGGT-3'	5'-TGGCCAGGATAATAGGGACA-3'
Dnajc5	5'-CTCTATGTGGCGGAGCAGTT-3'	5'-GCTGTATGACGATCGGTGTG-3'
Poldip3/Skar	5'-AGTACAGGATGCCAGGGAGA-3'	5'-GGAGAACAGGAGCGGTGTAG-3'
Madd	5'-CTGAGCTAGGCGGTGAGTTCCCT-3'	5'-GTACTTGTGGCTCACCACTTCTTTA-3'

S1 Table: List of synthetic oligonucleotides and PCR primers.

(A) Synthetic oligonucleotides used to construct the U6 snRNA expression plasmid. (B) PCR primers used to detect transcripts and small RNAs. † denotes a commercially available primer included in the Mir-X First Strand Synthesis Kit (TaKaRa).
