# Supplementary figures and images for: U6 snRNA expression prevents toxicity in TDP-43-knockdown cells
Source: PLoS One. 2017 Nov 10;12(11):e0187813. doi: 10.1371/journal.pone.0187813 (PMC5681290; doi:10.1371/journal.pone.0187813)

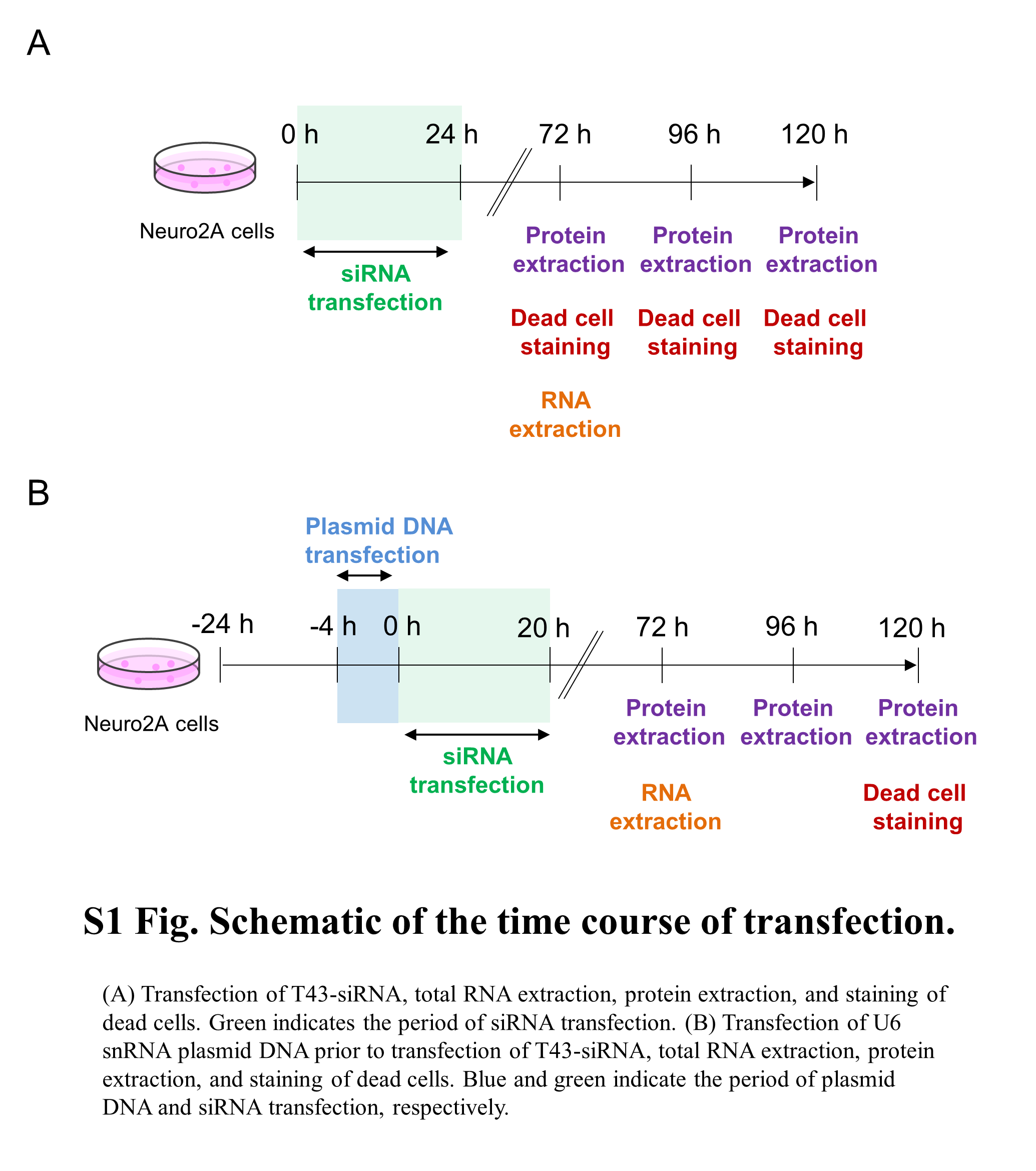

Supplement: S1 Fig — (A) Transfection of T43-siRNA, total RNA extraction, protein extraction, and staining of dead cells. Green indicates the period of siRNA transfection. (B) Transfection of U6 snRNA plasmid DNA prior to transfection of T43-siRNA, total RNA extraction, protein extraction, and staining of dead cells. Blue and green indicate the period of plasmid DNA and siRNA transfection, respectively. (TIF) [file pone.0187813.s002.tif]

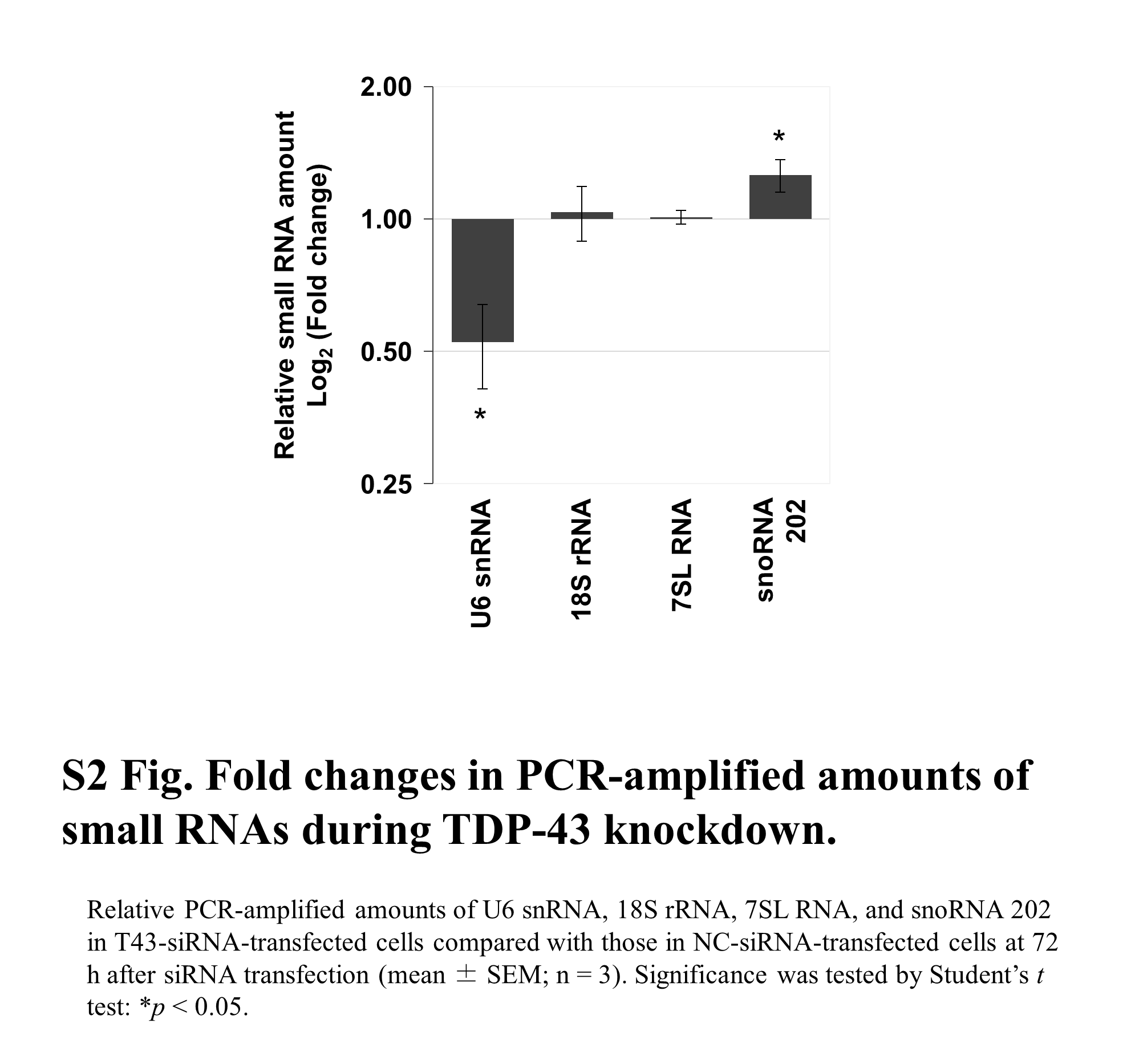

Supplement: S2 Fig — Relative PCR-amplified amounts of U6 snRNA, 18S rRNA, 7SL RNA, and snoRNA 202 in T43-siRNA-transfected cells compared with those in NC-siRNA-transfected cells at 72 h after siRNA transfection (mean ± SEM; n = 3). Significance was tested by Student’s t test: *p < 0.05. (TIF) [file pone.0187813.s003.tif]

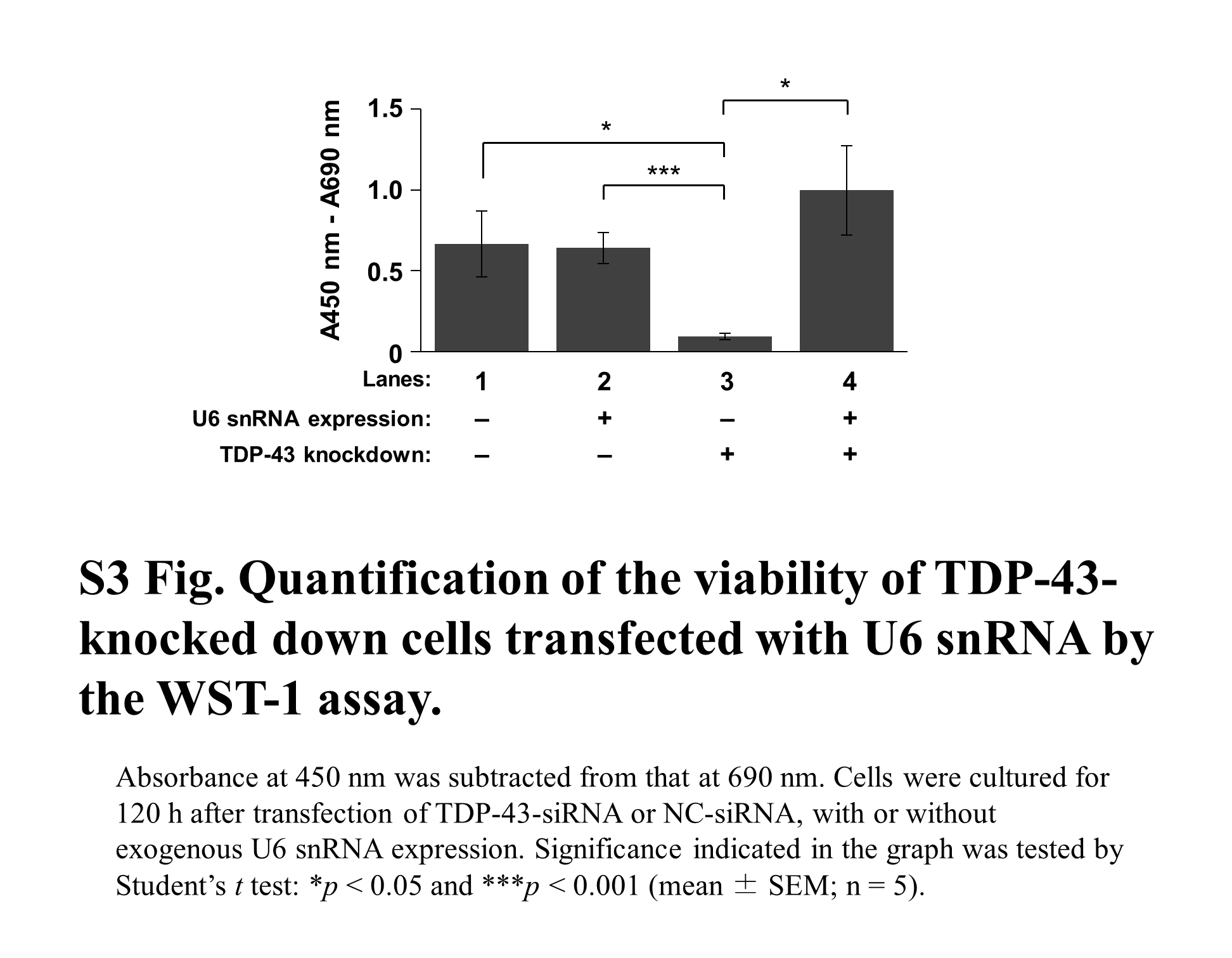

Supplement: S3 Fig — Absorbance at 450 nm was subtracted from that at 690 nm. Cells were cultured for 120 h after transfection of TDP-43-siRNA or NC-siRNA, with or without exogenous U6 snRNA expression. Significance indicated in the graph was tested by Student’s t test: *p < 0.05 and ***p < 0.001 (mean ± SEM; n = 5). (TIF) [file pone.0187813.s004.tif]

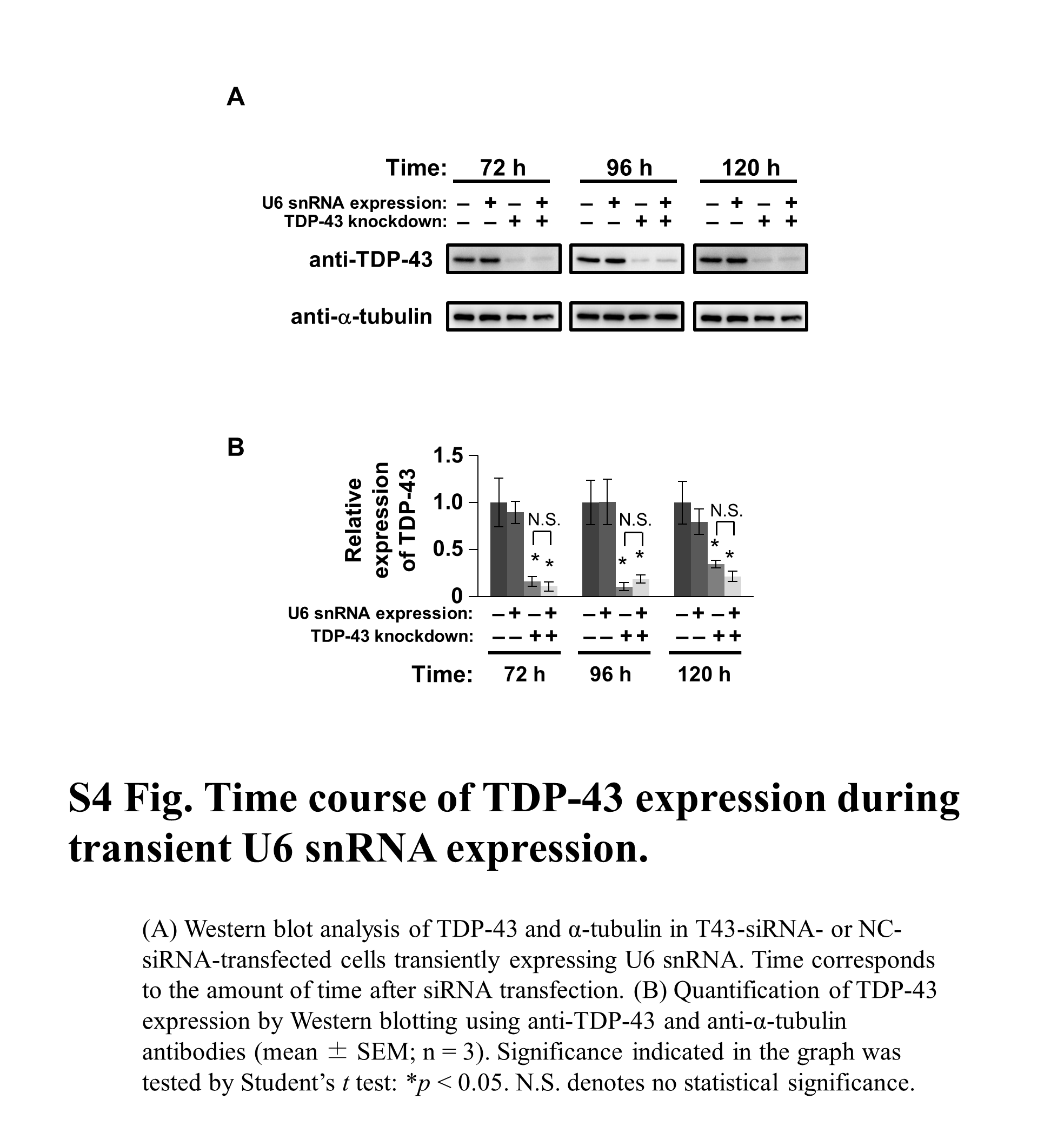

Supplement: S4 Fig — (A) Western blot analysis of TDP-43 and α-tubulin in T43-siRNA- or NC-siRNA-transfected cells transiently expressing U6 snRNA. Time corresponds to the amount of time after siRNA transfection. (B) Quantification of TDP-43 expression by Western blotting using anti-TDP-43 and anti-α-tubulin antibodies (mean ± SEM; n = 3). Significance indicated in the graph was tested by Student’s t test: *p < 0.05. N.S. denotes no statistical significance. (TIF) [file pone.0187813.s005.tif]

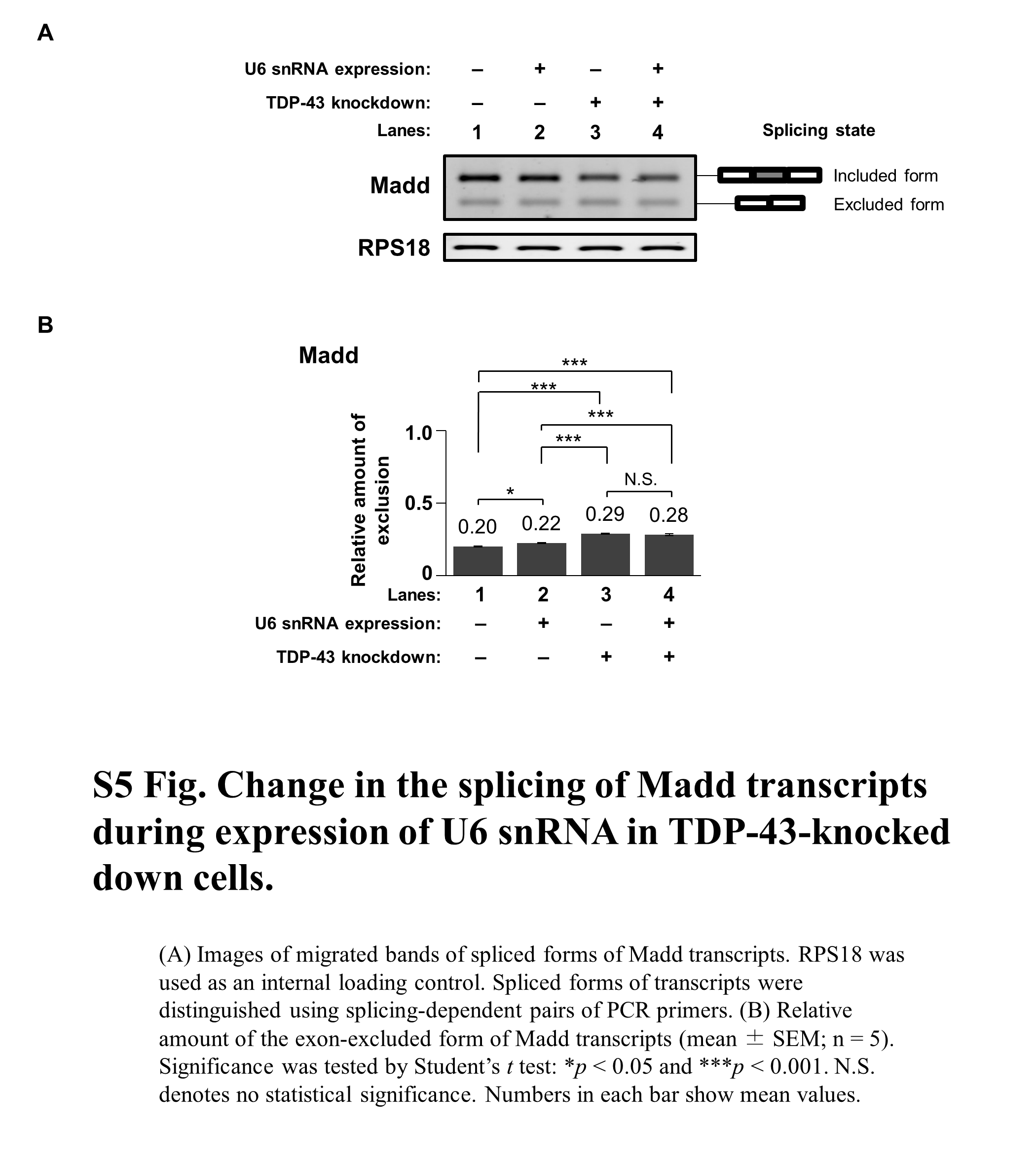

Supplement: S5 Fig — (A) Images of migrated bands of spliced forms of Madd transcripts. RPS18 was used as an internal loading control. Spliced forms of transcripts were distinguished using splicing-dependent pairs of PCR primers. (B) Relative amount of the exon-excluded form of Madd transcripts (mean ± SEM; n = 5). Significance was tested by Student’s t test: *p < 0.05 and ***p < 0.001. N.S. denotes no statistical significance. Numbers in each bar show mean values. (TIF) [file pone.0187813.s006.tif]
